# Characteristics of clinical trials in rare vs. common diseases: A register-based Latvian study

**DOI:** 10.1371/journal.pone.0194494

**Published:** 2018-04-03

**Authors:** Konstantins Logviss, Dainis Krievins, Santa Purvina

**Affiliations:** 1 Department of Pharmacology, Riga Stradins University, Riga, Latvia; 2 Department of Education and Science, Pauls Stradins Clinical University Hospital, Riga, Latvia; 3 University of Latvia, Riga, Latvia; AUSTRIA

## Abstract

**Background:**

Conducting clinical studies in small populations may be very challenging; therefore quality of clinical evidence may differ between rare and non-rare disease therapies.

**Objective:**

This register-based study aims to evaluate the characteristics of clinical trials in rare diseases conducted in Latvia and compare them with clinical trials in more common conditions.

**Methods:**

The EU Clinical Trials Register (clinicaltrialsregister.eu) was used to identify interventional clinical trials related to rare diseases (n = 51) and to compose a control group of clinical trials in non-rare diseases (n = 102) for further comparison of the trial characteristics.

**Results:**

We found no significant difference in the use of overall survival as a primary endpoint in clinical trials between rare and non-rare diseases (9.8% vs. 13.7%, respectively). However, clinical trials in rare diseases were less likely to be randomized controlled trials (62.7% vs. 83.3%). Rare and non-rare disease clinical trials varied in masking, with rare disease trials less likely to be double blind (45.1% vs. 63.7%). Active comparators were less frequently used in rare disease trials (36.4% vs. 58.8% of controlled trials). Clinical trials in rare diseases enrolled fewer participants than those in non-rare diseases: in Latvia (mean 18.3 vs. 40.2 subjects, respectively), in the European Economic Area (mean 181.0 vs. 626.9 subjects), and in the whole clinical trial (mean 335.8 vs. 1406.3 subjects). Although, we found no significant difference in trial duration between the groups (mean 38.3 vs. 36.4 months).

**Conclusions:**

The current study confirms that clinical trials in rare diseases vary from those in non-rare conditions, with notable differences in enrollment, randomization, masking, and the use of active comparators. However, we found no significant difference in trial duration and the use of overall survival as a primary endpoint.

## Introduction

Rare diseases are heterogeneous life-threatening or seriously debilitating conditions that affect less than one person in 2 000 individuals across the European Union (EU) [1]. Most rare disease patients suffer from ultra-orphan diseases, with a prevalence of less than 1 per 50 000 persons [2]. Development of medicinal products intended for the treatment, diagnosis or prevention of rare diseases (orphan drugs) can be very challenging due to distinct rare disease features, such as small patient populations, low event rates, inadequate understanding of disease natural course, and a lack of previous clinical trials [3]. The most obvious challenge in rare disease trials is the recruitment of the right patients in adequate numbers [4–6], therefore multicenter and multinational collaboration is often required.

Drug approval is usually based on a phase III, double blind, randomized, controlled trial (RCT) widely regarded as the gold standard. However, it may be particularly difficult to use phase III trial design for evaluating drugs intended to treat rare diseases. For example, phase III clinical trials supported efficacy for 45% of orphan drug, compared to 73% of non-orphan drug, US Food and Drug Administration (FDA) approvals for oncological indications [7]. Some orphan drugs were granted marketing approval by the FDA and the European Medicines Agency (EMA) without randomized, doubled blind, placebo controlled pivotal trials [8], but on the basis of uncontrolled phase II trial, retrospective study, or a literature analysis [9].

Most of the EMA approved orphan drugs demonstrated moderate overall quality of clinical evidence [10, 11]. The majority of the drugs were tested in trials involving fewer than 200 patients and lasting less than two years [12]. Nearly half of the studies applied some type of blinding [13] and used placebo as a comparator [12]. RCT are available for approximately 60% of orphan drugs authorized in the EU [9, 11–14]. Duration of orphan drug trials is often too short in relation to the natural history of the disease [9, 12, 15]. Dose finding studies and the use of active comparators are frequently lacking.

An analysis of ClinicalTrials.gov compared interventional clinical trials in rare against non-rare diseases [16]. Rare disease studies differed to non-rare disease studies across all characteristics that were examined. Rare disease trials enrolled fewer patients, were longer and more likely to be early phase, non-randomized, single arm, and open label. A higher proportion of rare disease trials were multicenter and multinational studies, included pediatric patients, and were terminated early. However, rare diseases consist of heterogeneous groups of conditions, which differ in their incidence (ranging from low to relatively high), survival (short vs. long), and treatment response (small vs. large) [4]. Therefore, clinical trial designs may vary among different rare diseases. For example, pivotal studies supporting the EMA granted marketing authorizations of orphan drugs consisted of populations ranging from as few as 7–12 patients to several hundred patients [4, 13, 14]. Marketing authorizations for oncological orphan drugs were mostly granted on the basis of large studies in relatively common disorders, whereas most of those for genetic diseases were based on much smaller studies [4].

Oncology is the major therapeutic area for orphan drugs [17, 18]. Moreover, prevalence of oncological rare diseases is often higher than that of many non-oncological rare conditions [11]. It makes oncology a specific rare disease group with a particular interest. Nevertheless, pivotal trials of orphan drugs approved by the FDA for treatment of cancer related indications involved less participants, were less likely to be randomized and double blind, but more frequently used surrogate primary endpoints and more treated patients had serious adverse events, compared with pivotal trials used to approve non-orphan cancer drugs [19]. Similarly, pivotal clinical evidence submitted to the EMA for marketing authorization of oncological orphan drugs was often limited by low patient numbers, inadequate follow-up, and lack of randomization or blinding [11]. Although, RCT data were provided in 57% of the studies. Another study, focusing on non-cancer orphan drugs approved by the FDA, found that orphan drugs had fewer pivotal clinical trials and fewer participants, but development times, proportions with randomization, blinding, and use of placebo and clinically relevant endpoints were similar between orphan and non-orphan drugs [20]. In neurological diseases, all drugs approved by the FDA without an orphan indication included at least two double blind RCT, compared to 32% of drugs with an orphan indication [8]. Though, 74% of orphan drugs had at least one such trial. Additionally, orphan drugs had less pivotal trials per drug and smaller trial sizes than non-orphan drugs.

Therapies for lysosomal storage disorders (mostly enzyme replacement therapies) were approved by the FDA mainly based on small clinical trials, with either surrogate or biomarker endpoints (e.g. in Gaucher disease, Fabry disease, and cystinosis) [21]. Identification of the most appropriate primary endpoint and target population of a pivotal clinical trial is crucial for successful marketing approval of orphan drugs [22]. Ideally, if appropriate hard clinical endpoint exists, it should be preferred [3, 23]. However, if the clinically meaningful (hard) primary endpoint (such as overall survival) is chosen, the small sample size or limited trial duration may not be adequate to demonstrate statistical significance [13, 22]. When the use of hard clinical endpoints is impossible or impractical, then surrogate endpoints can be considered, but need to be fully justified [3, 23]. A number of drugs for the treatment of rare diseases have been approved using surrogate endpoints, which are generally often used in clinical studies of orphan drugs, in contrast to quality of life (QoL) related endpoints and hard clinical endpoints. For example, in the EU, less than 30% of orphan drug pivotal studies included a QoL-related endpoint and less than 20% of the studies used at least one hard primary endpoint [13]. Especially for some ultra-rare disorders, surrogate endpoints are a necessary part of drug development process [24].

Apparently, limited data from pivotal trials of orphan drugs, coupled with usually high drug costs, may create obstacles in reimbursement and market access of these products [11, 17, 25]. Though, in Belgium, orphan drugs are more likely to be reimbursed despite lower quality of clinical evidence [15]. Latvia is known to be a small market with restricted availability and accessibility of orphan drugs [17, 25]. However, clinical trials in rare diseases have not been studied there. Clinical studies can allow rare disease patients access to investigational drugs, while the quality of data from these studies may affect reimbursement decisions and further market access of rare disease therapies. The current study aims to evaluate the characteristics of clinical trials in rare diseases conducted in Latvia and compare them with clinical trials in more common conditions.

## Materials and methods

### EU Clinical Trials Register

We used the EU Clinical Trials Register (clinicaltrialsregister.eu) to identify clinical trials related to rare diseases and to compose a control group of clinical trials in non-rare diseases for further comparison of the trial characteristics. The register contains information on interventional clinical trials on medicines conducted in the EU, or the European Economic Area (EEA), which started after 1 May 2004. The present study was performed in May 2016, covering a period of 12 years. The EU Clinical Trials Register provides the public with information held in the EU clinical trials database (EudraCT). The EudraCT database is maintained by the EMA and used by the national competent authorities to enter clinical trial data, originally provided by the sponsor, and to support supervision of clinical trials.

### Search strategy

Advanced search tools (filters) were used to restrict the search to clinical trials related to rare diseases which were conducted in Latvia. The search filters used included: “Country—Latvia”, “Rare disease”, and “Investigational medicinal product (IMP) with orphan designation in the indication”. A total of 51 clinical trials with a unique EudraCT number, which identifies the trial throughout its lifespan, were identified (S1 Appendix). The detailed trial protocol-related data were accessed through the Organization for Standardization (ISO) code for Latvia (LV). Data displayed for some clinical trials were incomplete or contained inconsistencies. For the missing information of such trials, we used data provided by other EEA countries (via the clinicaltrialsregister.eu) and/ or ClinicalTrials.gov (a clinical trials database maintained by the US National Library of Medicine at the National Institutes of Health). The following characteristics of the trials were analyzed: primary endpoints, randomization, masking, comparators, estimated trial enrollment and duration.

### Control group

For the control group of clinical trials in non-rare diseases, 376 unique clinical studies in common conditions conducted in Latvia were initially classified by therapeutic areas and trial phases. Then, 102 clinical trials were randomly chosen to compose the control group (S2 Appendix). Ratio of the control group clinical trials to rare disease clinical trials was 2:1. Proportions of therapeutic areas and trial phases were maintained between the two groups for comparability reasons. Therapeutic areas of clinical trials in the control group were distributed as follows: oncology—40 trials (39.2%); infections—20 trials (19.6%); endocrine and metabolic diseases—18 trials (17.6%); nervous system—6 trials (5.9%); blood diseases—6 trials (5.9%); circulatory system—4 trials (3.9%); respiratory system—4 trials (3.9%); and digestive system—4 trials (3.9%). 66 clinical trials (64.7%) were phase III trials, 28 (27.5%) were phase II trials, and 8 (7.8%) were phase IV trials.

### Primary endpoints

We analyzed whether overall survival (OS) was used as one of the primary endpoints in clinical trials. Outcome measures other than OS were classified as non-OS. Examples of such endpoints included disease-specific mortality, morbidity, clinical events, hospitalization, patient reported outcomes (symptoms, functioning, health-related QoL), physical signs, laboratory measures, biomarkers, radiological tests, response rates, progression-free survival (PFS), disease-free survival (DFS), pharmacokinetic (PK) parameters, and adverse events (AE). Only the primary endpoints were evaluated; secondary endpoints were not taken into account.

### Comparators

Controls (comparators) were classified into the following types: placebo, different (active) treatment, different dose or regimen of the study drug (dose comparison), no treatment, or external (historical) control [26].

### Data analysis

We used Fisher's exact test for statistical analysis of categorical variables: primary endpoints, randomization, masking, and comparators. T-test was used for scalar values: estimated trial duration and enrollment (S3 Appendix). 5% was used as a significance level of the tests, considering that with p<0.05 the null hypothesis could be rejected.

## Results

### Clinical trials in rare diseases

A total of 51 interventional clinical trials related to rare diseases, which were conducted in Latvia, were identified through the EU Clinical Trials Register (Table 1). 28 trials (54.9%) involved IMP with orphan designation in the studied indication. A total of 35 unique IMP were studied in 29 different rare conditions. Oncology was the biggest therapeutic area, with 20 clinical trials (39.2%), followed by infections, with 10 trials (19.6%), and endocrine and metabolic diseases, with 9 trials (17.6%). Multidrug-resistant tuberculosis (MDR-TB) was the most studied condition, with 7 trials (13.7%), followed by chronic lymphocytic leukemia (CLL), with 4 trials (7.8%), and chronic myelogenous leukemia (Ph+ CML), acromegaly, and pseudomonas aeruginosa infection in cystic fibrosis, with 3 trials (5.9%) in each condition. 33 clinical trials (64.7%) were phase III trials (including two phase II/III trials), 14 (27.5%) were phase II trials, and 4 (7.8%) were phase IV trials.

**Table 1:** Clinical trials in rare diseases.

INN (trade name/ code name)	Condition	Trial design	Comparator	Primary endpoint	Estimated duration (months)	Estimated enrollment (number of subjects)		
						Latvia	EEA	Whole CT
Bedaquiline (Sirturo)	MDR-TB	Phase II, RCT, double blind	Placebo	SCC	58	15	15	150
Bedaquiline (Sirturo)	MDR-TB	Phase II, open label		SCC	40.5	13	23	225
Bedaquiline (Sirturo)	MDR-TB	Phase III, RCT, double blind	Placebo	SCC	66	7	13	600
Delamanid (Deltyba)	MDR-TB	Phase II, RCT, double blind	Placebo	SCC; PK; AE	10	13	26	201
Delamanid (Deltyba)	MDR-TB	Phase II, open label extension		AE	14	80	100	430
Delamanid (Deltyba)	MDR-TB	Phase II, open label		AE; PK	17	20	30	30
Delamanid (Deltyba)	MDR-TB	Phase III, RCT, double blind	Placebo	SCC	59	60	150	390
Dopastatin (BIM-23A760)	Acromegaly	Phase II, open label		GH levels	8	10	24	24
Dopastatin (BIM-23A760)	Acromegaly	Phase II, open label		GH and IGF-1 levels	14	5	60	80
Dopastatin (BIM-23A760)	Carcinoid syndrome	Phase II, open label		Symptom relief (diarrhea and/ or flushes)	17	10	60	80
Tobramycin (TOBI Podhaler)	Pseudomonas aeruginosa infection in CF	Phase III, RCT, double blind	Placebo	FEV1	12	6	40	100
Tobramycin (TOBI Podhaler)	Pseudomonas aeruginosa infection in CF	Phase III, open label extension		AE	12	6	40	100
Tobramycin (TOBI Podhaler)	Pseudomonas aeruginosa infection in CF	Phase III, open label extension		AE	9	3	40	100
Meropenem (Meronem)	Severe acute necrotizing pancreatitis	Phase IV, RCT, double blind	Placebo	Development of pancreatic or peripancreatic infection	27	40	240	240
Somapacitan (NNC0195-0092)	Growth hormone deficiency	Phase III, RCT, double blind/ open label	Placebo (double blind), Somatropin (open label)	Truncal fat percentage	38.6	3	66	280
Claudiximab (IMAB362)	Gastric/ esophageal cancer	Phase II, open label		Rate of remission	17	8	25	30
Claudiximab (IMAB362)	Gastric/ esophageal cancer	Phase II, RCT, open label	EOX (epirubicin, oxaliplatin, capecitabine)	PFS; AE	41	65	85	231
Lanreotide (Somatuline)	Acromegaly	Phase IV, open label		Injection intervals (6 or 8 weeks) based on IGF-1 levels	24	20	110	150
Lanreotide (Somatuline)	Carcinoid syndrome	Phase IV, RCT, double blind	Placebo	Usage of s/c octreotide as rescue medication to control symptoms (diarrhea and/ or flushing)	36	4	60	100
Recombinant microbial lipase (SLV339)	Exocrine pancreatic insufficiency due to chronic pancreatitis	Phase II, RCT, double blind	Placebo	CFA; CNA; stool parameters; nutritional parameters; clinical symptomatology; AE	6	30	60	80
Temozolomide (Temodal)	Glioblastoma multiforme	Phase III, RCT, open label	Dose comparison (conventional vs. dose-intensive temozolomide)	OS; PFS	48	40	834	834
Catumaxomab (Removab)	Malignant ascites	Phase II/III, RCT, open label	Paracentesis	Puncture-free survival	21	36	168	216
Ovarian cancer vaccine (CVac)	Epithelial ovarian cancer	Phase II, RCT, double blind/ open label	Placebo (double blind), SOC (open label)	OS	60	15	244	286
Somatropin (Somatropin Biopartners)	Growth hormone deficiency	Phase III, RCT, open label	Somatropin (daily Genotropin)	Height velocity; AE	29	5	134	144
Teplizumab (MGA031)	Recent-onset type 1 diabetes mellitus	Phase II/III, RCT, double blind	Placebo	Total daily insulin dose; HbA1c levels	36	25	385	530
Bosutinib (Bosulif)	Ph+ CML	Phase III, RCT, open label	Imatinib (Glivec)	Complete cytogenetic response rate	108	30	206	412
Bosutinib (Bosulif)	Ph+ CML	Phase III, open label extension		AE (with special focus on diarrhea); BCR-ABL mutations; OS	84	2	136	500
Ciprofloxacin DPI (BAYQ3939)	Non-CF bronchiectasis	Phase III, RCT, double blind	Placebo	Frequency of pulmonary exacerbations	34	28	200	400
Ciprofloxacin DPI (BAYQ3939)	Non-CF bronchiectasis	Phase III, RCT, double blind	Placebo	Frequency of pulmonary exacerbations	28	28	172	400
Duvelisib (IPI-145)	CLL/SLL	Phase III, RCT, open label	Ofatumumab (Arzerra)	PFS	72	22	174	307
Duvelisib (IPI-145)	CLL/SLL	Phase III, open label extension	Ofatumumab (Arzerra)	Overall response rate	24	22	174	307
Pazopanib (Votrient)	Renal cell carcinoma	Phase III, RCT, double blind	Placebo	PFS	24	10	175	400
Pazopanib (Votrient)	Renal cell carcinoma	Phase III, open label extension		AE	24	3	98	145
Sildenafil (Revatio)	Pulmonary arterial hypertension	Phase IV, RCT, double blind	Dose comparison (1/5/20 mg tid)	6MWT	29	5	82	219
Paclitaxel, micellar (Paclical)	Ovarian/ peritoneal/ fallopian tube cancer	Phase III, RCT, open label	Paclitaxel, Cremophor EL (Taxol)	CA-125 levels; PFS; hypersensitivity reactions	48	25	350	650
Eprodisate disodium (Kiacta)	AA amyloidosis	Phase III, RCT, double blind	Placebo	CrCl; SCr; progression to end-stage renal disease	40	10	119	280
Obinutuzumab (Gazyvaro)	CLL	Phase III, open label		AE	55	7	560	800
Eltrombopag (Revolade)	ITP	Phase II, RCT, double blind	Placebo	Platelet count	18	10	129	422
Dinaciclib (SCH-727965)	CLL	Phase III, RCT, open label	Ofatumumab (Arzerra)	PFS	38	8	225	466
Lapatinib (Tyverb)	Squamous cell carcinoma of the head and neck	Phase III, RCT, double blind	Placebo	DFS	27	4	422	680
Tivantinib (ARQ 197)	Non-small cell lung cancer	Phase II, open label extension		AE	24	1	4	10
Masitinib (AB1010)	Mastocytosis	Phase III, RCT, double blind	Placebo	Symptom relief (pruritus, flushes, depression, and asthenia)	42	15	170	200
Octocog alfa (BAY 81–8973)	Hemophilia A	Phase III, open label		Annualized number of bleeds	51	2	50	75
Clazosentan (AXV-034343)	Aneurysmal subarachnoid hemorrhage	Phase III, RCT, double blind	Placebo	Cerebral vasospasm-related morbidity; all-cause mortality	21	15	620	1146
Brivaracetam (Briviact)	Focal epilepsy/ POS	Phase III, RCT, double blind	Placebo	POS (type I seizures) frequency	43	40	350	900
Brivaracetam (Briviact)	Focal epilepsy/ POS	Phase III, open label extension		AE	68	40	274	720
Nilotinib (Tasigna)	Ph+ CML	Phase III, open label		Rate of molecular response	48	8	743	806
Ibandronic acid (Bondronat)	Multiple myeloma	Phase III, RCT, open label	Zoledronic acid (Zometa)	Skeletal related events	36	25	424	424
Turoctocog alfa (NovoEight)	Hemophilia A	Phase III, open label extension		Frequency of development of FVIII inhibitors	90	8	36	215
Fingolimod (Gilenya)	Multiple sclerosis in pediatric patients	Phase III, RCT, double blind	IFN β-1a (Avonex)	Annualized relapse rate	111	4	82	190
Pegylated recombinant human hyaluronidase (PEGPH20)	Pancreatic ductal adenocarcinoma	Phase III, RCT, double blind	Placebo	PFS; OS	47	24	224	420

INN, international nonproprietary name; CT, clinical trial; EEA, European Economic Area; MDR-TB, multidrug-resistant tuberculosis; RCT, randomized controlled trial; SCC, sputum culture conversion; PK, pharmacokinetics; AE, adverse events; GH, growth hormone; IGF-1, insulin-like growth factor-1; CF, cystic fibrosis; FEV1, forced expiratory volume in one second; PFS, progression-free survival; s/c, subcutaneous; CFA, coefficient of fat absorption; CNA, coefficient of nitrogen absorption; OS, overall survival; SOC, standard of care; HbA1c, hemoglobin A1c (glycated hemoglobin); Ph+ CML, Philadelphia chromosome positive chronic myelogenous leukemia; DPI, dry powder for inhalation; CLL, chronic lymphocytic leukemia; SLL, small lymphocytic lymphoma; tid, three times a day; 6MWT, six-minute walk test; CA-125, cancer antigen 125; CrCl, creatinine clearance; SCr, serum creatinine; ITP, immune (idiopathic) thrombocytopenic purpura; DFS, disease-free survival; POS, partial onset seizure; FVIII, coagulation factor 8; IFN β-1a, interferon beta-1a.

### Characteristics of clinical trials in rare vs. common diseases

**Fig 1:**
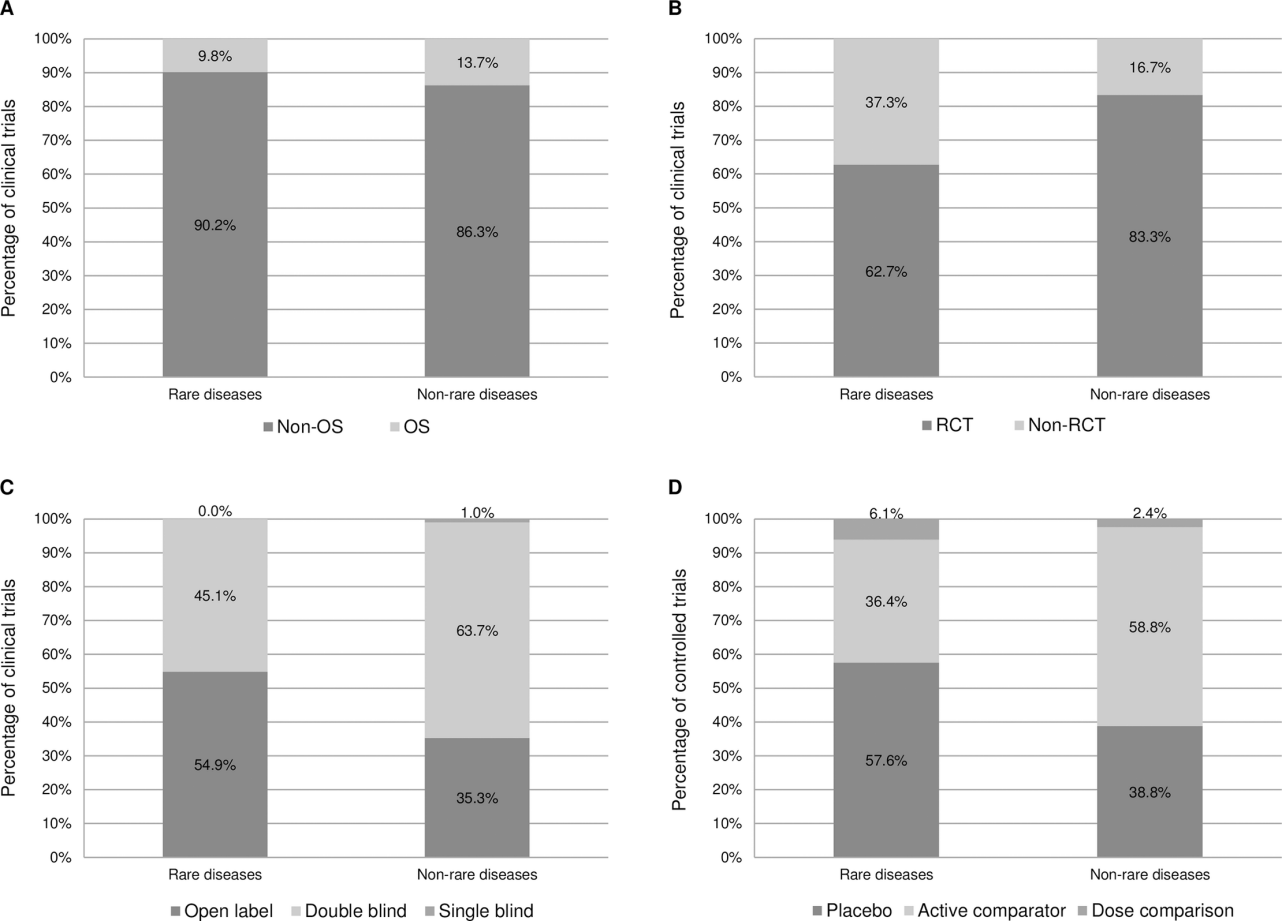
Characteristics of clinical trials in rare vs. non-rare diseases. (A) Primary endpoints. (B) Randomization. (C) Masking. (D) Comparators.

**Fig 2:**
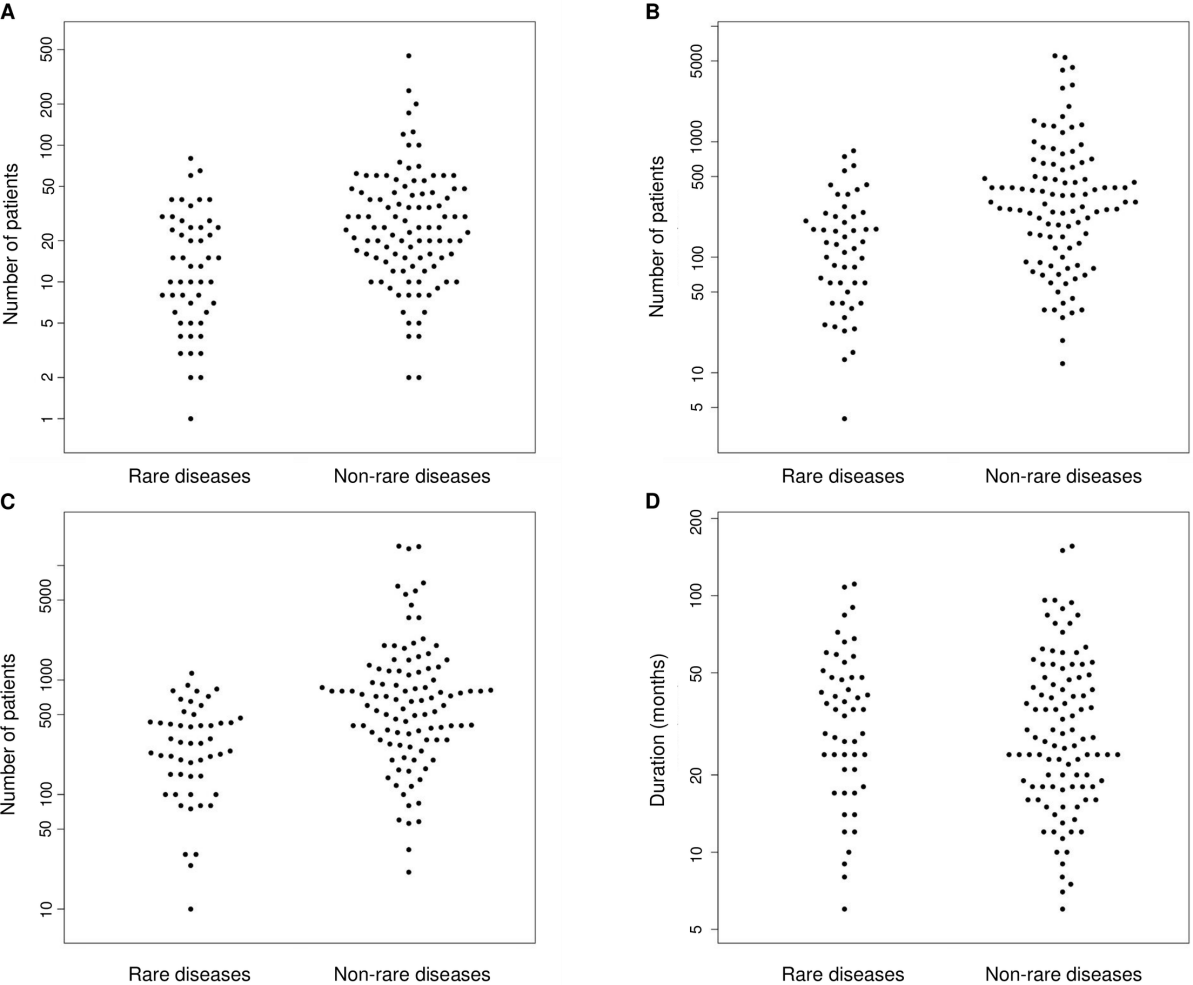
Estimated trial enrollment and duration. (A) Enrollment in Latvia. (B) Enrollment in the EEA. (C) Enrollment in the whole clinical trial. (D) Trial duration.

We found no significant difference in the use of OS as a primary endpoint in clinical trials between rare and non-rare diseases (9.8% vs. 13.7%, respectively; p = 0.608) (Fig 1A). However, clinical trials in rare diseases were less likely to be randomized controlled trials (62.7% vs. 83.3%; p = 0.008) (Fig 1B). Rare and non-rare disease clinical trials varied in masking, with rare disease trials less likely to be double blind (45.1% vs. 63.7%; p = 0.035) (Fig 1C). Active comparators were less frequently used in rare disease trials (36.4% vs. 58.8% of controlled trials; at a significance level of 10%, as Fisher's exact test p = 0.052) (Fig 1D). Clinical trials in rare diseases enrolled fewer participants than those in non-rare diseases: in Latvia (mean 18.3 vs. 40.2 subjects; 95% confidence interval (CI) of the difference 9.8–33.9; p = 0.014) (Fig 2A), in the EEA (mean 181.0 vs. 626.9 subjects; 95% CI 239.3–652.5; p<0.001) (Fig 2B), and in the whole clinical trial (mean 335.8 vs. 1406.3 subjects; 95% CI 548.0–1593.0; p<0.001) (Fig 2C). Although, we found no significant difference in trial duration between the groups (mean 38.3 vs. 36.4 months; 95% CI -10.9–7.1; p = 0.652) (Fig 2D). All studies included in the analysis were multicenter and multinational trials involving multiple EEA member states and/ or being conducted both within and outside the EEA.

## Discussion

According to Lithuanian study published in 2008, shortly after joining the EU, the number of clinical trials aimed at orphan drugs remained low in the Baltic States [27]. Between May 2004 and June 2007, four clinical trials on orphan medicinal products were approved in Lithuania, one trial in Estonia, and no trials in Latvia. The current study covered a period of time between May 2004 and May 2016 and included both orphan drugs and non-orphan drugs for rare diseases. As a result, 51 clinical trials in rare diseases were identified in Latvia. More than half of them (28 trials) involved orphan medicinal products, indicating that the number of clinical trials for orphan drugs has notably increased in recent years. It should be pointed out, however, that the principal investigators of all of the studies described in the current analysis were not from Latvia, but centers in our country provided sites/ patients to these trials. In fact, none of the trial sponsors was from Latvia or other Baltic States. This applied to both, clinical trials in rare and non-rare diseases, which were almost exclusively sponsored by global commercial pharmaceutical companies. There was only one non-commercial sponsor in each group.

The majority of rare disease clinical studies were phase III studies, while oncology was the biggest therapeutic area, followed by infections and endocrine and metabolic diseases. Oncological conditions and metabolic and endocrine disorders are generally the main indications of orphan drugs [17, 18]. The finding that infectious diseases made the second largest therapeutic area in our study can be explained by the fact that MDR-TB was the most studied condition. In fact, the three Baltic States are classified as MDR-TB high burden (high priority) countries with the highest prevalence of MDR-TB in the EU/EEA [28]. In addition, these countries have established high quality surveillance systems to monitor drug resistance. Disease prevalence as well as diagnostic and treatment options of rare diseases may vary between different EU countries. In this context, conducting clinical studies in MDR-TB in the Baltic States seems rational, as appropriate patients are concentrated there in relatively high numbers.

Our findings are consistent with the previous studies reporting that RCT are available for approximately 60% of rare disease therapies [9, 11–14] and that significant differences exist in enrollment, randomization, blinding, and the use of active comparators between clinical trials in rare and non-rare conditions [8, 11, 15, 16, 19]. As might be expected, clinical trials in rare diseases recruited fewer participants. This is in line with the recent investigation by Hee et al. [29], who examined the association between the disease prevalence and sample size for interventional clinical trials in rare diseases and found that trials of rarer diseases were noticeably smaller than the less rare diseases trials (generally sample size increases as prevalence increases). The authors were surprised that a majority of trials were conducted in one country only, regardless of the disease prevalence, given the opportunity to recruit more patients in multinational studies. Although, Bell and Tudur Smith [16] found that a higher proportion of rare disease trials were multicenter and multinational studies compared to non-rare disease studies. In the current analysis, all clinical trials were multinational studies involving multiple EEA member states and/ or being conducted both within and outside the EEA. One might logically expect longer trials in rare diseases (as found by Bell and Tudur Smith [16]) to compensate for few participants in order to demonstrate statistical significance, but this was not confirmed in the current study. One might also expect more sophisticated statistical modeling, which does not seem to be true empirically [30]. Unkel et al. reviewed the methods used to evaluate therapies in two rare conditions (paediatric multiple sclerosis and Creutzfeldt-Jakob disease) and found that the statistical methodology used was fairly basic. This applied in particular to paediatric multiple sclerosis, for which the evidence on therapeutic interventions was almost exclusively based on observational studies. Studies of this type might have special importance for rare diseases, as large sample size is not readily available for trials in these conditions [6, 14, 31], though observational studies were out of the scope of the current analysis, which was aimed at interventional studies only.

The current study has certain limitations. Firstly, we analyzed all (completed and ongoing) interventional clinical trials related to all rare diseases and orphan drugs (authorized and not authorized). In contrast, most previous studies (except the analysis of ClinicalTrials.gov [16]) were restricted to specific therapeutic areas, such as oncology or neurology, and/ or assessed only pivotal clinical trials (primarily supporting efficacy) of authorized orphan drugs [7, 8, 11, 13, 19–21, 32]. Secondly, this is a register-based study. Bell and Tudur Smith carried out the US register-based analysis of ClinicalTrials.gov [16] (the work extended later by Hee et al. [29], but without comparison between rare and non-rare disease trials), described a number of limitations of the dataset and pointed out that other registers, such as clinicaltrialsregister.eu, can also be used. In the EU Clinical Trials Register, a trial protocol reports the estimated enrollment, rather than the actual number of patients recruited. The expected numbers of patients to be enrolled in clinical trials may be overestimated [29]. For example, in the above mentioned analysis of ClinicalTrials.gov [16], the actual enrollment in rare disease trials was 70.1% of the anticipated enrollment, compared to 81.6% in non-rare disease trials.

The EU Clinical Trials Register contains information on clinical trials, which started after May 2004, while the orphan drug regulation 141/2000 was introduced in the EU in 2000 [1]. Trials started before the implementation of the clinical trial directive 2001/20/EC in 2004 [33] are not listed in the register. Moreover, in March 2011, version 8.0 of the EudraCT database was launched putting in place a more comprehensive set of validation rules for data entry. Historical data, entered into the database between May 2004 and March 2011, may be incomplete or contain inconsistencies, due to less stringent requirements for data entry, or absence of some fields in earlier versions of EudraCT. In addition, research and regulatory procedures alter over time. Potentially less rigorous evaluation criteria might have been used for older therapies than for recently approved ones. Information can also be missing because data have not been provided by the sponsor. However, these limitations seem to apply equally to both, rare and non-rare disease clinical trials, and are not likely to cause a bias.

## Conclusions

Quality of clinical evidence is affected by numerous challenges faced by investigational drugs for rare diseases. Despite the fact that RCT are available for over 60% of rare disease therapies, clinical trials in rare diseases vary from those in non-rare conditions. Clinical studies in orphan diseases enroll fewer participants and are less likely to use randomization, blinding, and active comparators. However, we found no significant difference in trial duration and the use of overall survival as a primary endpoint.

## Supporting information

S1 Appendix Clinical trials in rare diseases. (XLSX)

S2 Appendix Clinical trials in non-rare diseases. (XLSX)

S3 Appendix Data analysis. (XLS)
